# Isothiazolinone Biocides: Chemistry, Biological, and Toxicity Profiles

**DOI:** 10.3390/molecules25040991

**Published:** 2020-02-23

**Authors:** Vânia Silva, Cátia Silva, Pedro Soares, E. Manuela Garrido, Fernanda Borges, Jorge Garrido

**Affiliations:** 1 CIETI/School of Engineering (ISEP), Polytechnic of Porto, 4200-072 Porto, Portugal; vfmsi@isep.ipp.pt (V.S.); emg@isep.ipp.pt (E.M.G.); 2 CIQUP/Department of Chemistry and Biochemistry, Faculty of Sciences, University of Porto, 4169-007 Porto, Portugal; catia.soares@fc.up.pt (C.S.); pedro_hgsoares@hotmail.com (P.S.)

**Keywords:** biocides, isothiazoles, isothiazolinones, chemistry, biological/toxicity relationships, stability, analysis

## Abstract

The importance of isothiazole and of compounds containing the isothiazole nucleus has been growing over the last few years. Isothiazolinones are used in cosmetic and as chemical additives for occupational and industrial usage due to their bacteriostatic and fungiostatic activity. Despite their effectiveness as biocides, isothiazolinones are strong sensitizers, producing skin irritations and allergies and may pose ecotoxicological hazards. Therefore, their use is restricted by EU legislation. Considering the relevance and importance of isothiazolinone biocides, the present review describes the state-of-the-art knowledge regarding their synthesis, antibacterial components, toxicity (including structure–activity–toxicity relationships) outlines, and (photo)chemical stability. Due to the increasing prevalence and impact of isothiazolinones in consumer’s health, analytical methods for the identification and determination of this type of biocides were also discussed.

## 1. Introduction

**Figure 1:**
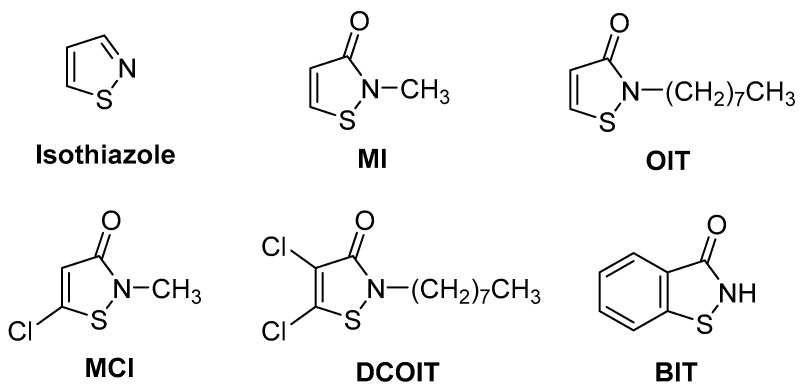
Chemical structures of isothiazole and of the most frequently used isothiazolinone biocides in consumer products.

Organic compounds containing five-membered heterocyclic rings play an important role in many industrial sectors. Among them, isothiazole (Figure 1) and its derivatives have found applications in different fields since they present useful biological properties, such as antimicrobial, antibacterial, antifungal, antiviral, anticancer, and anti-inflammatory activities [1]. Furthermore, isothiazoles have been described to act as inhibitors of proteases for the treatment of anxiety and depression, as inhibitors of aldose reductase, and as 5-hydroxytryptamine receptor antagonists [1]. Chemically, isothiazole (1,2-thiazole) is a five-membered heteroaromatic that is considered to be derived from thiophene, in which the second position is occupied by a nitrogen atom (Figure 1) [2].

The noticeable biological effects observed for isothiazole-containing compounds have generated an enormous interest on this scaffold for drug discovery and development programs, which resulted in a steady increase in the number of related patent applications as well as in the successful introduction of isothiazole-based derivatives to the market. Among them, the most extensively used for industrial applications and reactive intermediates, for the synthesis of various organic substances including pharmaceuticals and agrochemicals, are those based on isothiazolin-3-one (isothiazolinone).

Isothiazol-3-ones are known for their remarkable antifungal and antibacterial properties, being extensively used as biocides in a variety of industrial water treatment applications for the control of microbial growth and biofouling. They have also been recommended as preservatives to prevent fungal growth in a wide range of manufactured goods, such as emulsion paints, wood varnishes, adhesives, and natural and artificial leather [3]. The isothiazolinones most commonly found in commercial applications, alone or in combination, are methylisothiazolinone (MI), methylchloroisothiazolinone (MCI), benzisothiazolinone (BIT), octylisothiazolione (OIT), and dichlorocthylisothiazolinone (DCOIT) (Figure 1). Methylisothiazolinone is commonly used in wastewater treatment processes [4], cosmetics, paints, and detergents [5], and in combination with MCI (in proportions of 3:1) as an active ingredient of the commercial biocide, Kathon [6]. Although BIT and OIT are forbidden to be used in cosmetics [7], they are usually applied in cleaning and leather products, respectively [5], and as antifouling coating agents. DCOIT, the biocidal ingredient in SeaNine 211, is a widely used antifouling agent to deter the undesirable biofouling phenomenon [6,7,8]. 

Although there has been an increased use of isothiazolinones over the last years, concerns related to their inherent sensitization potential and allergic contact dermatitis, frequently observed both in consumers as well as workers in various industries, have been reported [5,9,10]. Moreover, cross-reactivity between different isothiazolinones has been demonstrated in animal assays and broadens the potential consequences of becoming sensitized to this type of compound [9].

As a result of the widespread use of this type of heterocycles and the renewed interest in this scaffold, this review provides an overview of the most used isothiazolinone ring-containing compounds. In-depth information on the synthesis of the most frequently isothiazolinone biocides used in consumer products and on biological/toxicity profile is presented. Additionally, given the increased concern about the possible unintended side effects that isothiazolinones could have on human health and the environment, this review also covers chemical and photochemical stability issues and the analytical procedures often used for their determination.

## 2. Synthesis of Isothiazolinones

Isothiazolinones are well-known biocides that are used as additives in a wide range of industrial products. Since obtaining the first derivatives at the end of the 19th century, hundreds of protocols describing their synthesis emerged in the literature. A simple search in Scifinder discloses a considerable number of reports concerning the synthesis of the isothiazolinone derivatives MI, MCI, BIT, OIT, and DCOIT (Figure 1) spawned between patents and peer-reviewed journals. 

### 2.1. Synthesis of the Isothiazolinone Derivatives MI, MCI, OIT, and DCOIT

**Scheme 1:**
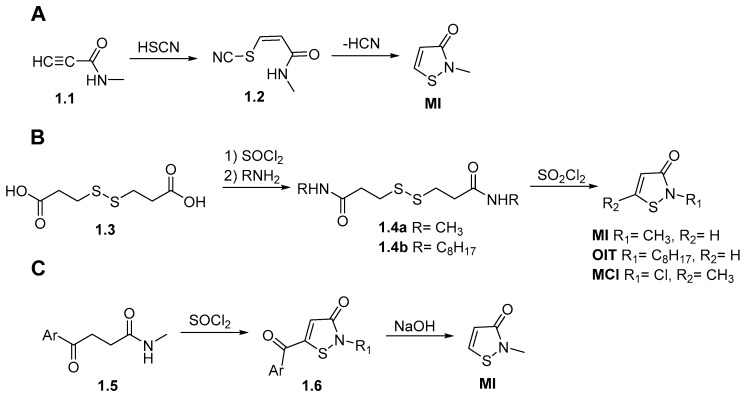
Synthesis of the biocides methylisothiazolinone (MI), methylchloroisothiazolinone (MCI), and octylisothiazolinone (OIT) as described in references [11,12,13]. (**A**) Synthesis of MI; (**B**) Synthesis of MI and OIT by the one-step chlorination-cyclization of their respective 3,3′-dithiopropionamides; (**C**) Alternative route for the synthesis of MI.

A number of protocols can be found in the literature describing the synthesis of MI, MCI, and OIT biocides from the cyclization of either thioacrylamides or 3,3′-dithiopropionamides (Scheme 1) [11,12,13,14]. 

The first synthesis of 2-methylisothiazol-3(2H)-one (MI) was reported by Crow and Leonard in 1964 (Scheme 1A) [11]. The authors described the synthesis of MI through the cyclization of cis-*N*-methyl-3-thiocyanatoacrylamide (**1.2**), which was conveniently prepared from its precursor *N*-methylpropiolamide (**1.1**), with an overall yield of 80%. 

Later on in 1971, the Szamborsky group described the synthesis of MI and OIT by the one-step chlorination–cyclization of their respective 3,3′-dithiopropionamides (**1.4a** and **1.4b**), which were readily prepared by amidation, via acyl chlorides, of the commercially available 3,3′-dithiodipropionic acid (**1.3**) (Scheme 1B) [12]. The overall yields obtained for MI and OIT were 33% and 96%, respectively. Additionally, in the same paper, the authors also described obtaining MCI as a side product of the cyclization reaction that gave MI. However, despite registering a yield below 20% for MCI, the authors did not describe any efforts toward the optimization of the reaction conditions in order to increase the yield of this compound. Indeed, only in 2003, Clerici et al. reported the first efforts to optimize the yield of MCI in the reaction initially reported by the Szamborsky group [14]. Through the handling of the ratio between the amide precursor and the chlorinating agent (SO_2_Cl_2_), the reaction temperature and the time of reaction Clerici et al. were capable of increasing the yield of MCI to 34%.

Tsolomitis and Sandris in 1987 reported an alternative route for the synthesis of MI (Scheme 1C) [13]. The authors initially promoted the formation of 4-benzoyl-2-methylisothiazol-3(2H)-one (**1.6**) through the reaction of *N*-methyl-4-oxo-4-phenylbutanamide (**1.5**) and SOCl_2_. Then, the final compound was obtained, in 90% yield, after the nucleophilic displacement of the benzoyl group from compound **1.6**.

**Scheme 2:**
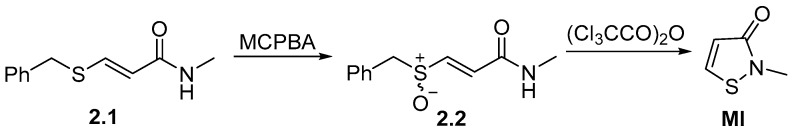
Synthesis of the biocide MI as described in reference [15].

In 1994, Beeley et al. published the first and only synthesis of MI that, unlike most of the previously described routes, did not rely on SO_2_Cl_2_ to induce the formation of the isothiazol-3-one ring (Scheme 2) [15]. In their work, the authors initially obtained the sulfoxide derivative (**2.2**) from the corresponding (*Z*)-3-(benzylsulfinyl)-*N*-methylpropenamide (2.1). Then, using trichloroacetic anhydride, the cyclization of sulfoxide **2.2** was induced and the final compound MI was obtained. Notably, the proposed protocol avoided the formation of chlorinated by-products such as MCI, providing MI with a yield close to 70%.

**Scheme 3:**
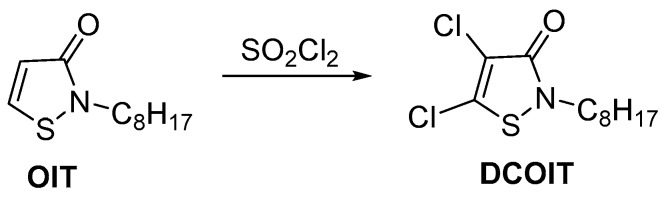
Synthesis of the biocide dichlorocthylisothiazolinone (DCOIT) as described in reference [16].

The synthesis of DCOIT was described by Weiler and collaborators in 1977 (Scheme 3) [16]. DCOIT was successfully obtained from a reaction that occurred between OIT and SO_2_Cl_2_, with a 51% yield.

Most of the synthetic approaches presented that are related with the synthesis of isothiazol-3-ones MI, MCI, OIT, and DCOIT start from the cyclization of amides that are readily prepared from cheap carboxylic acid precursors available from a wide range of chemical suppliers. However, despite their established industrial application, only a handful of protocols describing their synthesis have been reported. In summary, it seems that at least for MI, MCI, OIT, and DCOIT, most of the synthetic strategies are only found in industrial patents that due to the protection of intellectual property provide minimal details about the synthetic conditions and the purity of the final compounds.

### 2.2. Synthesis of 1,2-Benzisothiazol-3(2H)-one (BIT)

**Scheme 4:**
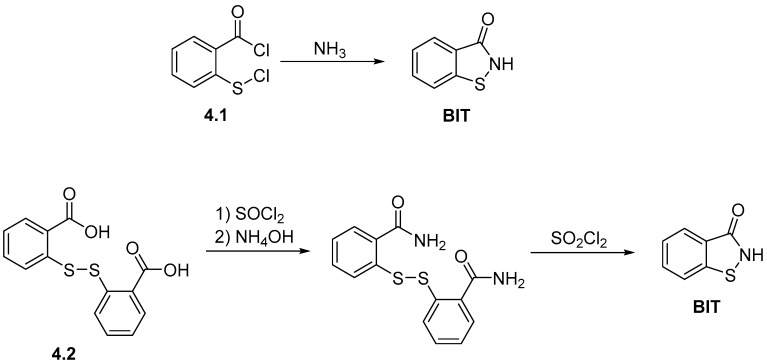
Synthesis of the biocide benzisothiazolinone (BIT) as described in references [18,19,20].

The first reported synthesis of benzisothiazolinone (BIT) dates from 1923, and since then several authors have contributed to expand the available methodologies to obtain this compound [17]. The traditional approaches to obtain BIT are centered on the condensation of 2-(chlorosulfanyl)benzoyl chloride (**4.1**) with ammonia and on the halogenation, amidation, and intramolecular cyclization processes using 2,2′-dithiodibenzoic acid (**4.2**) as starting reagent (Scheme 4) [18,19,20].

However, despite the usefulness of these protocols to readily access BIT, their application has been restricted due to the use of harsh reaction conditions, low yields, and the use of highly toxic and corrosive reagents such as chlorine or SO_2_Cl_2_. Hence, in the last 20 years, several methodologies to obtain BIT, circumventing the previously described limitations, have been published in the literature. Herein, we present some of the most relevant examples of such protocols.

**Scheme 5:**
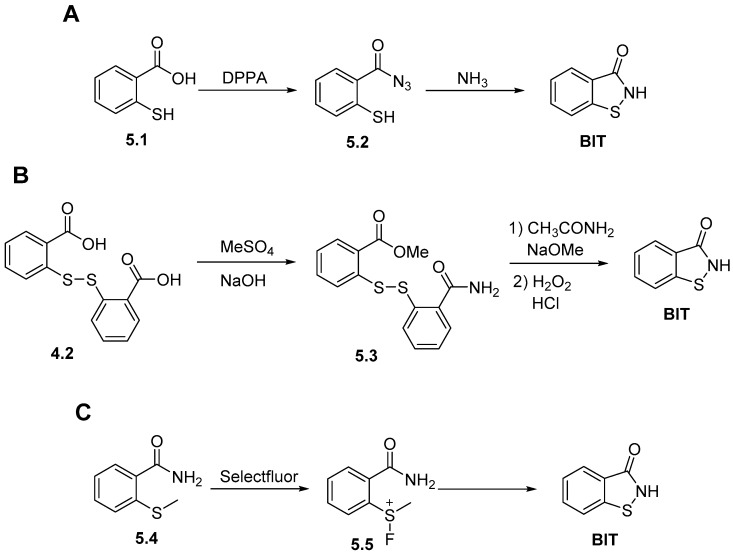
Synthesis of the biocide BIT as described in (**A**) reference [21], (**B**) reference [22], and (**C**) reference [23].

The Kajiwara group in 2000 published one of the first chlorine-free syntheses of BIT [21]. The authors prepared in situ the acyl azide (**5.2**) from the reaction between diphenyl phosphoryl azide (DPPA) and the starting material 2-mercaptobenzoic acid (**5.1**) (Scheme 5A). Then, by gently warming the reaction from 0 °C to room temperature, the authors avoided the Curtius rearrangement and promoted the formation of an S–N bond via nucleophilic substitution that furnished BIT in 81% yield. In 2003, Jin et al. also described the synthesis of BIT (reaction yield 90%) from an one step amidation-cyclization reaction of 2,2′-dithiodibenzoate (**5.3**), which was easily obtained from the commercially available 2,2,-dithiodibenzoic acid (**4.2**) (Scheme 5B) [22]. Recently, in 2018, Yang et al. used selectfluor to obtain BIT from 2-(methylthio)benzamide (**5.4**) (Scheme 5C) [23]. In this protocol, the authors efficiently used selectfluor to form in situ a fluorosulfonium salt (**5.5**) and then obtained the final compound, in 80% yield, via N–H bond formation followed by C–S bond cleavage.

**Scheme 6:**
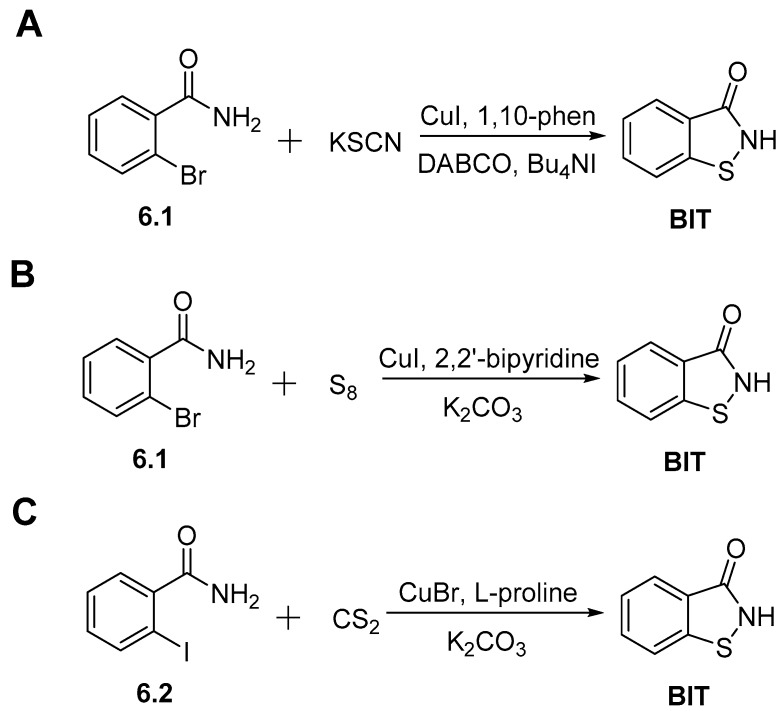
Synthesis of the biocide BIT using copper catalysts as described in (**A**) reference [24], (**B**) reference [25], and (**C**) reference [26].

In the last decade, a series of copper-catalyzed reactions emerged as alternative synthetic strategies to obtain BIT [24,25,26]. In these reactions, copper catalysts combined with the appropriate ligands were used to catalyze the formation of a C–S bond between 2-halobenzamides and sulfur sources, followed by an intramolecular S–N bond formation to afford the final compound. For instance, in 2012, the Xi group described the synthesis of BIT from the reaction between 2-bromobenzamide (**6.1**) and potassium thiocyanate (KSCN) in the presence of CuI, 1,10-phenantroline, 1,4-diazabicyclo[2.2.2]octane (DABCO), and the phase transfer agent Bu_4_NI (Scheme 6A), obtaining an overall yield of 60% [24]. In 2013, Krasikova and Katkevics obtained BIT from 2-bromobenzamide (**6.1**) and sulfur (S_8_) in the presence of CuI and 2,2′-bipyridine, with a yield below 20% (Scheme 6B) [25]. Chen’s group in 2016 used CuBr and L-proline as a ligand to catalyze the reaction between 2-iodobenzamide (6.2) and CS_2_ (Scheme 6C), and obtained BIT in high yields (>75%) without the use of a phase transfer agent [26].

In summary, over the last 30 years, considerable efforts have been made to increase the diversity of the synthetic portfolio to obtain BIT and at the same time avoid the use of the very corrosive and toxic reagents employed on the most traditional reactions to obtain this compound. However, the continuous interest on 1,2-benzisothiazol-3(*2H*)-one biocide capacity and medicinal properties make us believe that in the coming years, the available portfolio to obtain BIT and its derivatives could be further enriched with novel reactions increasing the substrate scope of these reactions and provide greener reactions without the use of metal catalysts and using environmentally friendly solvents.

## 3. Antibacterial Action and Toxicity of Isothiazolinones

**Scheme 7:**
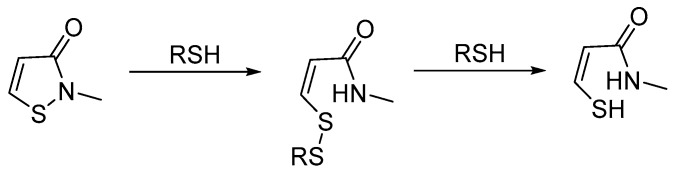
Reaction pathway for the interaction of isothiazolinones and thiols from cellular components (e.g., glutathione (GSH)) [29].

As referred previously, isothiazolinone derivatives MI, MCI, BIT, OIT, and DCOIT are powerful biocides that are used as preservatives in a wide range of daily life products, such as detergents, paints, and cosmetic products [27]. These compounds were described to be able to diffuse across the bacterial cell membrane and the cell wall of fungi. In the intracellular media, the electron-deficient sulfur of the N–S bond of these compounds can react with the nucleophilic groups of the cellular components, such as the thiols from cysteines of proteins active sites blocking their enzymatic activity and ultimately causing cellular death [28]. Indeed, as demonstrated by Collier et al., MI, MCI and BIT are able to readily react with thiol-containing compounds, such as glutathione (GSH) and cysteine, to form disulfide derivatives (Scheme 7) and promote a series of reactions that impair key cellular functions, causing cellular growth inhibition in a few minutes and cellular death in few hours [29].

The antibacterial and antifungal activity of isothiazolinones is highly valued by a number of industries. Therefore, over the years, several reports have been prepared illustrating their biocidal activity against a wide spectrum of industrial biological contaminants. For instance, Collier et al. evaluated the biocidal activity of MI, MCI, and BIT against *Schizosacchurornyces pombe* and *Escherichia coli* (Table 1) [30]. In this study, MI showed the highest minimum growth inhibitory concentration (MIC) values, 245 μg/mL and 41 μg/mL for *S. pombe* and *E. coli*, respectively. On the other hand, MCI displayed the lowest MIC values of the tested compounds, 2.6 μg/mL and 0.5 μg/mL for *S. pombe* and *E. coli*, respectively. These results highlighted that the presence of the chlorine in the double bond of the isothiazolinone ring improves the compound’s biocidal activity.

In 1993, Green compared the efficacy of BIT and a commercial formulation containing a mixture of MCI:MI (4:1) against an autofluorescent species of *Legionella bozemanii* [31]. The biocide containing the mixture of MCI:MI was capable of inhibiting *Legionella* film formation below the assay detection levels at concentrations as low as 50 ppm of formulation, whereas BIT needed concentrations above 200 ppm to present a similar effect. Furthermore, the content of MCI (% *w*/*v*) in the MCI:MI formulation was much lower than the amount of active compound on the commercial BIT formulation. Hence, the overall results of this study revealed a higher potency of MCI against *Legionella*. In a recent study, Rushton et al. demonstrated that BIT and a mixture of MI:MCI (3:1) displayed a bactericidal activity against 82 out of a panel 83 strains of bacteria from *Burkholderia cepacia* complex, which are well-known industrial contaminants [32]. Two independent studies compared the biocide activity of MI, MCI, OIT, DCOIT, and a mixture of MCI/MI against *Aspergillus niger* and *Saccharomyces cerevisiae* (Table 2) [33,34]. The highest MIC and minimum biocidal concentration (MBC) values for both fungi were disclosed after MI treatment. On the other hand, all the other biocides showed a high inhibitory capacity with MIC and MBC values below 1 mg/L against both fungi. Importantly, the authors observed that the potency of MCI, OIT, DCOIT, and of the MCI/MI mixture was nearly identical.

In addition, Robson’s group reported that OIT was able to protect plasticized polyvinyl chloride (pPVC) samples from the colonization of some fungus present in forest and grassland soil [35], and in another study, Hu’s group demonstrated that leather samples pre-treated with OIT were protected from *Staphylococcus aureus* contamination [36].

Despite being widely used in a plethora of industrial products due to their relevant biocidal effect, over the last decades, several publications described isothiazolones’ potential health hazards to both industrial workers and costumers. Indeed, there are several reports associating these compounds to severe dermatitis and to the impairment of pulmonary functions [5,37,38,39,40,41,42,43,44,45,46,47,48,49,50]. 

A number of reports linked allergic contact dermatitis derived from isothiazolinones exposure to the activation of inflammatory mediators in skin cells [51,52,53]. Conversely, the first studies about the toxicity of isothiazolinones inhalation to human respiratory systems were only released in the last years. In 2019, Yang’s group demonstrated that alveolar epithelial cells (MLE-12 cells) treated with a mixture of MI/MCI presented high levels of pro-apoptotic proteins such as BAX-Bcl-2 and cleaved caspase-3. Furthermore, in the same study, the authors observed that MI/MCI led to the release of pro-inflammatory cytokines such as TNF-α and IL-1β through the upregulation of the mitogen-activated protein kinases (MAPK) signaling pathway [54]. More recently, another group demonstrated that MI was capable of inducing cellular death and the activation of pro-inflammatory responses in bronchial epithelial cells (BEAS-2B cells). Additionally, the authors also signaled the possible carcinogenic effect of MI after gene profile analysis [55].

Recently, some works suggested a potential cytotoxicity effect of isothiazolinone preservatives on human liver and neuronal cell lines. For instance, Ranke’s group have studied the impact of MI, MCI, OIT, and DCOIT on glutathione metabolism and glutathione reductase activity in human liver (HepG2) cell lines (Table 3), showing that MCI and DCOIT were capable of significantly impairing cellular thiol reduction potential and inducing morphological changes in the cells, matching the ones observed during cellular necrosis [56]. Later, the same group compared the toxicological effect and the ecotoxicological effect of the same biocides using the HepG2 cell line, marine bacterium *Vibrio fischeri* cells, and green algae *Scenedesmus Vacuolatus*. In this study, the authors observed that MCI was the most toxic of the four compounds; however, the half maximum effective concentration (EC_50_) observed for this biocide was comparable with the ones observed for OIT and DCOIT (Table 3) [57].

**Scheme 8:**
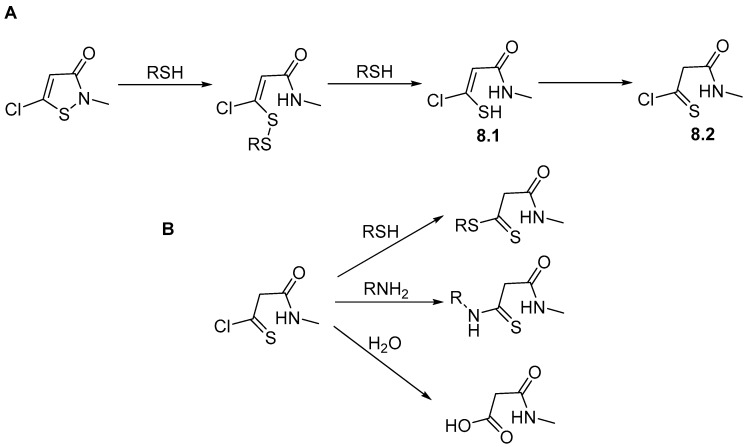
Reaction pathway of MCI. (**A**) Interaction with thiols from cellular components (e.g., GSH) and the formation of thio-acylchloride intermediates; (**B**) interaction of thio-acylchloride intermediates with thiols, amines, and water [29].

Additionally, the authors also observed that the toxicity of these compounds was not directly correlated with the increase of the compounds’ lipophilicity (Log Pow). Instead, as reported by Collier et al., it was found that the chlorine substituent at position 5 of the isothiazolinone ring increases the reactivity of the biocides toward thiols and the formation of the mercaptoacrylamide intermediate (**8.1**) by MCI ring opening, which can also contribute to the formation of the highly reactive thio-acylchloride intermediates (**8.2**) that are capable of interacting not only with thiols but also with amines, water, or other type of nucleophiles (Scheme 8) [29]. Therefore, despite increasing the biocidal effect of MCI, the formation of the acylating agent also increases the toxicity associated with this compound.

In a recent study, Gerhold’s group also alerted for the potential neurotoxic effect of OIT after observation of the negative impact of this biocide on the intracellular ATP levels of three neuronal cellular lines [58]. In 2014, Séralini’s group demonstrated that a BIT commercial formulation (Polysect Ultra) was cytotoxic against three human cell lines: JEG3 (placental), HEK293 (embrionic), and HepG2 (hepatic), highlighting that the biocide toxicity could go beyond its skin-sensitizing effect [59].

In summary, the biocidal activity of the reviewed isothiazolinones can be represented as MCI > OIT ≈ DCOIT > BIT > MI, and these results are paralleled by the cytotoxicity suggested for these compounds. As a consequence, despite their potency, the EU has adopted a strict regulation in order to balance the risks and benefits for the workers manipulating these biocides and for the final consumers of the industrial products preserved with these compounds (Table 4) [5,60]. However, under the light of the most recent toxicological data, it is expected that the EU could adopt a stricter regulation on the use of isothiazolinone biocides during industrial processes and suggest the replacement of the most toxic biocides for others with similar potency, but with a superior safety profile.

## 4. Stability of Isothiazolinones

The Biocidal Products Regulation (BPR, Regulation (EU) 528/2012) concerns the placing on the market and use of biocidal products, which are used to protect humans, animals, materials, or articles against harmful organisms such as pests or bacteria, by the action of the active substances contained in the biocidal product [61]. All biocidal products require an authorization before they can be placed on the market, and the active substances contained in that biocidal product must be previously approved. 

For active substance approval, data on stability must be provided, since evidence on how the quality of an active substance varies with time under the influence of a variety of environmental factors such as temperature, humidity, and light, and to establish a shelf-life for the product and recommended storage conditions. Moreover, information on the environmental fate and behavior of the active substance or biocidal product is required depending on the product type, i.e., the foreseen use and route of exposure. A detailed knowledge of the environmental fate characteristics of industrial biocides is critical to their safe use and protection for the environment.

Although the application of isothiazolinones is diverse, there are a few studies available in the literature regarding their stability and degradation. In general, isothiazolinone biocides present high volatility and are sensitive to thermal and pH conditions [62,63,64,65]. Literature data indicate that the stability of isothiazolinones in aqueous systems is limited being influenced by the presence, under environmental conditions, of nucleophiles, such as metals, amines, thiols, and sulfides [33,66,67]. Once this interaction occurs, the five-membered heterocyclic ring opens, and transformation/degradation occurs [62,67,68].

**Scheme 9:**
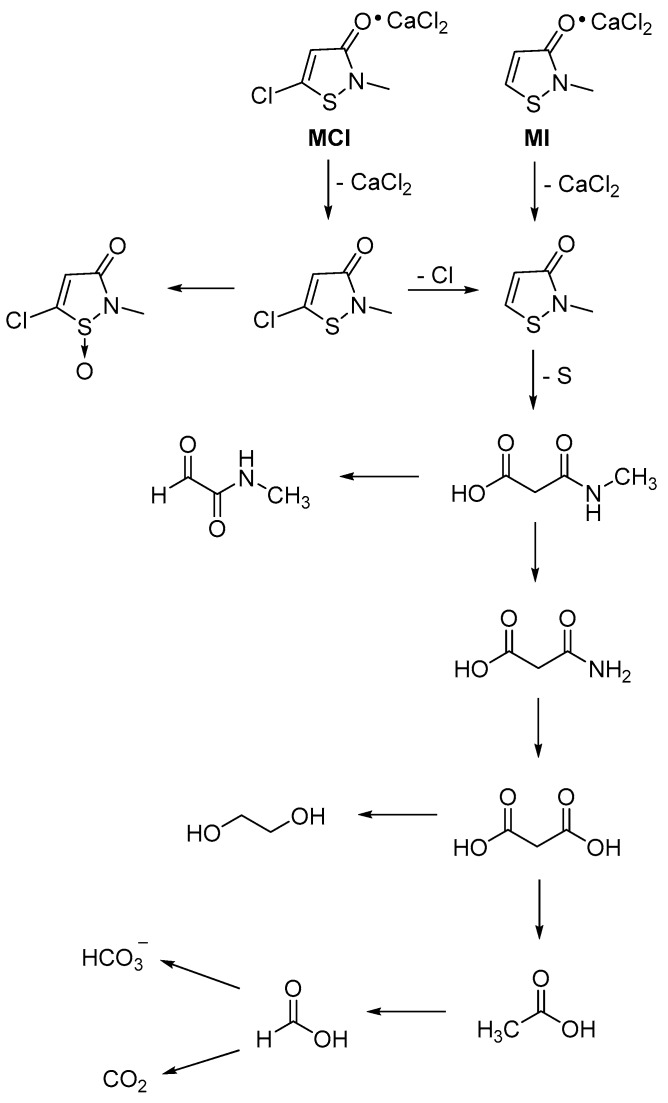
Major degradative pathway of MI and MCI [70].

Krzeminski et al. studied the modes and rates of dissipation of MI and MCI (calcium chloride salts) over a range of conditions likely to occur in the environment [69]. The degradation of both compounds at levels near 1 ppm was observed to occur readily by hydrolytic, photochemical, and biological action in the aquatic and terrestrial environments. From the data found, it was concluded that the rate of hydrolysis for MCI was greater with the increase of pH and temperature, and to only a limited extent, with the increasing ionic strength of the buffer. The compound is stable under acidic conditions, but the rate of disappearance increases by a factor of about 2000 in going from pH 4.5 to 11, and from 7 to 40°, the rate increases by 1–2 orders of magnitude. Moreover, it was observed that the rate of degradation of MCI by biological mechanisms is concentration-dependent, decreasing with increasing concentration. MI, which is less potent than MCI, is biologically degraded in river water or a simulated sewage treatment plant faster than MCI. Both compounds are degraded by UV radiation and show little adsorption by river silt but are readily absorbed and metabolized by aquatic ferns. According to the authors, the decomposition of both isothiazolones by several chemical and biological mechanisms ensures that the compounds will not persist in the environment and thus the use of these types of isothiazolones will not produce an undue ecological disturbance to the environment [69]. Following this work, the authors also sought to characterize, isolate, and identify the major transformation products of MI and MCI, in several environmental systems and, as a consequence, define their degradative pathway in the aquatic environment [70]. In eight systems, covering chemical, biochemical, and photochemical aspects of environmental degradation, their disappearance was rapid with both compounds generating, qualitatively and quantitatively, a similar distribution of degradation products. The principal degradative pathway involved the dissociation from CaCl_2_, ring opening, and loss of Cl and S, and led to *N*-methylmalonamic acid [70]. Then, the degradation proceeds through the formation of malonamic, malonic, acetic, and formic acids and their conversion to CO_2_. Other products along the degradative pathway were tentatively identified as 5-chloro-2-methyl-4-isothiazolin-1-oxide, *N*-methylglyoxylamide, ethylene glycol, and urea (Scheme 9) [70].

Similar results were obtained in a study involving the MCI degradation of commercial biocide formulations. In order to measure the effective lifetime and understand the mechanism of degradation, Barman and Preston investigated the stability of the active component, 5-chloro-2-methyl-4-isothiazolin-3-one (MCI) in Kathon 886 MW and Kathon MWC biocides in both acidic and alkaline solutions [71]. It was found that these biocides are stable in acidic media. However, the active component undergoes degradation in alkaline solutions, and the rate of degradation is faster with an increase in pH. A high stability in acidic medium and a linear relationship between hydroxyl ion concentration and the first-order rate constant in the alkaline range suggested that the chemical degradation of Kathon biocides results from hydrolysis of the chlorinated isothiazolone. Yet, no identification of degradation products was accomplished. Barman also studied the influence of temperature on the degradation of Kathon 886 MW and Kathon MWC biocides in aqueous media and in a metalworking fluid concentrate [72]. In aqueous media, the half-life values obtained ranged from less than two hours at pH 9.6 and 60 °C to 46 days at room temperature and pH 8.5. It was also found that the active component (MCI) is quite stable in a typical metalworking fluid concentrate at room temperature, with an estimated half-life of six months. However, MCI depletes rapidly in the same concentration at 40 °C and 60 °C, with apparent half-life values of only 12 and less than two days, respectively [70].

Park and Kwon performed hydrolysis and photolysis tests (pH 4, 7, and 9 at 25 °C) for two of the most widely used isothiazolinones—MCI and MI—in batch experiments to evaluate their stability in consumer products [6]. Although a slight decrease in the MCI concentration (7%–35%) was observed in the hydrolysis test at pH 9 and in the phototransformation test at pH 4 and 9, no noticeable decrease of MI concentration was observed under any test conditions [6]. At pH 9, the phototransformation rate constant was not significantly different from the hydrolysis rate constant at the same pH (0.038 d^−1^), indicating that the photolysis rate is much slower than the hydrolysis rate. The first-order photolysis rate constant was determined to be 0.019 d^−1^ for MCI at pH 4 [6].

**Scheme 10:**
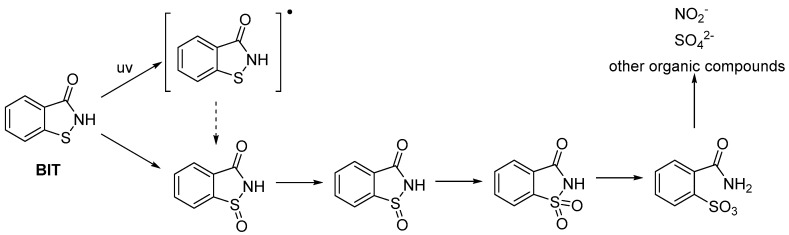
Proposed photolysis pathways of BIT under UV irradiation [75].

Benzisothiazolinone (BIT) has been described as being hydrolytically stable, presenting a half-life of more than 30 days in the environment [73]. BIT may be transported through soil and reach surface water, and it can still retain its biocidal qualities for 3 months when exposed to sunlight [73,74]. Wang et al. evaluated the photodegradation of BIT by UVC irradiation and verified the influence of several parameters, including initial BIT concentration, solution pH, and HCO_3_^−^ anion [75]. In acidic solutions (pH 5 and 6), the photodegradation rate of BIT was much slower than those in neutral (7 and 8) and basic solutions (pH = 10). The simulated pseudo-first-order kinetic at various pH indicated that the kinetic constant increased from 0.03 min^−1^ to 0.29 min^−1^ with an increase of pH from 5 to 8, and then decreased to 0.17 min^−1^ when a continuous increment of pH to 10 occurred. At pH 7 (20 mM phosphate buffering), the photodegradation under UVC irradiation can degrade BIT with a removal efficiency of 94.5%, at an initial BIT = 20 μM and 15 min irradiation time, compared with the negligible hydrolysis of BIT in dark conditions [75]. A slight enhancement of BIT photolysis was observed in the presence of (bi)carbonates. In this study, the identification of intermediates and products has been performed and based on the identified products, the photolysis pathway of BIT was proposed (Scheme 10).

**Scheme 11:**
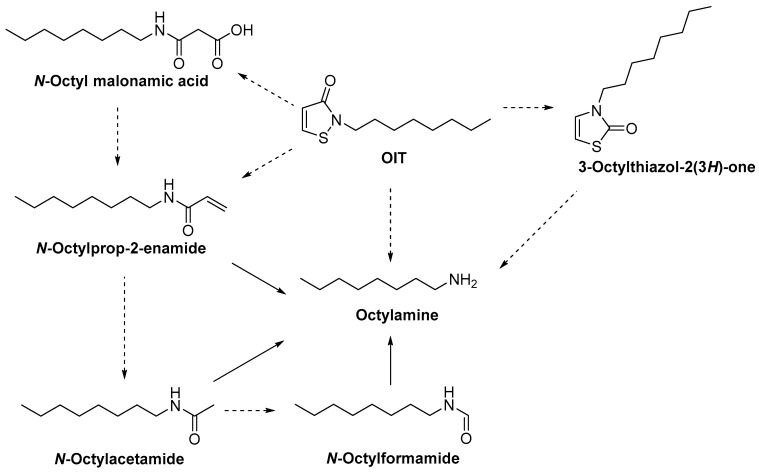
Photodegradation pathway proposed for OIT in water [76].

Octylisothiazolinone (OIT) is hydrolytically stable, showing a half-life time exceeding 40 days at 25 °C and pH 7.4 [57]. Bollmann et al. studied the leaching and fate of OIT used in facade coatings under natural conditions [76]. Kinetics experiments indicated that the photodegradation of OIT in tap water followed a first-order kinetics under the tested conditions, with a half-life of 28 h and a photodegradation rate constant of 0.026 h^−1^. Several phototransformation products were identified and validated by analytical standards. As described for other isothiazol-3(*2H*)-ones, the photodegradation of OIT is also initiated by a cleavage of the ring structure. In addition to 3-octylthiazol-2(*3H*)-one, resulting from OIT photoisomerization, the break of the N–S-bond led to the stepwise degradation of the ring originating the attainment of *N*-octyl malonamic acid, *N*-octylprop-2-enamide, *N*-octylacetamide, and *N*-octylformamide, as well as octylamine as a final product (Scheme 11) [76].

In another study, Chand et al. evaluated the biodegradation of OIT in raw wastewater [77]. A batch experiment was set-up in the laboratory with three conditions: anaerobic, aerobic with substrate (molasses), and aerobic without substrate. The results found showed that OIT is significantly biodegraded in all three conditions within 7 days. The half-life found for OIT was between 5 and 13 h for aerobic with substrate and anaerobic conditions, respectively [77].

**Scheme 12:**
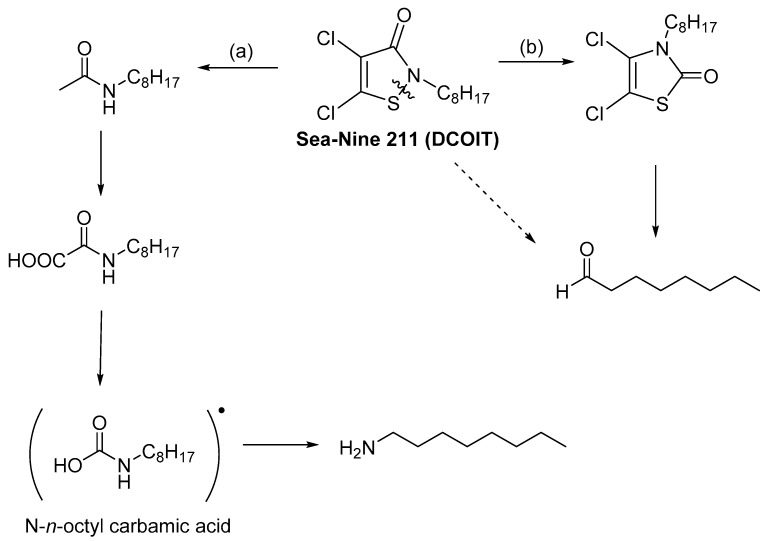
Photolytic degradation pathways and main phototransformation products of DCOIT (Sea-Nine 211) in aqueous environment [81].

Dichlorocthylisothiazolinone (DCOIT), the active ingredient in the commercial antifoulant SeaNine 211, has being fast degraded both biologically and chemically in natural seawater with reported half-lives of <1 day and <1 h for its removal from natural seawater and an aquatic microcosm, respectively [78,79]. However, contradicting results regarding its degradability have been reported [7]. Recently, Chen et al. investigated the degradation kinetics of DCOIT under different environmental conditions, such as pH, temperature, dissolved oxygen, sunlight, and marine organisms. In solutions buffered at pH 4, DCOIT degraded quickly within 30 d of observation, presenting a half-life of 6.8 d. In contrast, at pH 7, 98.4% of the dissolved DCOIT was degraded after 7 d, and the half-life was 1.2 d. At pH 9, DCOIT degraded gradually over the 30 d with an estimated half-life of 3.7 d [8]. The results showed that the degradation rate increased with increasing temperature. Half-lives of DCOIT at 4 °C, 25 °C, and 40 °C were >64 d, 27.9 d, and 4.5 d, respectively. Exposure to sunlight accelerated the degradation of DCOIT. The photolysis half-life of DCOIT was 6.8 d compared with 14.4 d for the dark control. No obvious biodegradation was observed for DCOIT after incubation for 4 d in natural seawater. A summary of Sea-Nine 211 half-lives in various environmental matrices can be found in the literature [7,79]. The phototransformation of Sea-Nine 211 in natural waters under natural sunlight increases following the order: lake water > river water > sea water > distilled water, showing a strong dependence on the composition of the irradiated media [80,81]. Simulated solar irradiation for 30 h resulted in a 97%, 92%, 87%, and 77% decline of Sea-Nine 211 concentration, respectively. Two main transformation pathways were proposed for the phototransformation of Sea-Nine 211 (Scheme 12) [81]. One of the pathways involves cleavage of the isothiazolone ring followed by subsequent dechlorination, hydroxylation, and oxidation yielding the formation of *N*-n-octyl malonamic acid, which is then decarboxylated to give the corresponding *N*-n-octyl acetamide [81]. Further oxidation of *N*-n-octyl acetamide leads to the formation of *N*-n-octyl oxamic acid, which could be then phototransformed to the corresponding *N*-n-octyl carbamic acid that undergoes subsequent decarboxylation, yielding n-octyl amine [81].

The second pathway (b) (Scheme 12) consists of the phototransposition of Sea-Nine 211, leading to 4,5-dichloro-3-n-octylthiazolin-2-one. The N–C alkyl bond cleavage of 4,5-dichloro-3-n-octylthiazolin-2-one and the oxidation of the alkyl group results in n-octanal [81]. During the photodegradation experiments, some other transformation products were detected but not identified [81].

In a recent contribution, Bollmann et al. studied whether biocidal compounds are easily degradable or persistent in a loamy sand soil, as typical for urban regions in Northern Europe. Different compound groups were tested including four isothiazolinones (MI, BIT, OIT, and DCOIT) [82]. More than 97% of the isothiazolinones were degraded during 120 days at the applied soil incubation conditions. The isothiazolinones degraded rapidly with half-lives below 10 days (MI 0.28 days, BIT 0.52 days, DCOIT 4.8 days, and OIT 9.3 days). The results obtained are in accordance with the literature available for MI and DCOIT.

## 5. Analysis and Determination of Isothiazolinone Biocides

The antimicrobial profile of isothiazolinones makes them highly efficient biocides, even at low concentrations. However, despite its effectiveness, some of them are strong sensitizers, producing skin irritation and allergies, and they could pose ecotoxicological risks [83]. Consequently, its use has been restricted by EU legislation to limited concentrations depending on the product type to be preserved (Table 4). In order to guarantee consumers’ health and ensure compliance to existing regulations, reliable methodologies must be used to identify and quantify this type of biocides.

Most of the commercial products containing isothiazolinone biocides include a broad variety of highly complex matrices that imply serious analytical difficulties. Thus, adequate sample preparation is a key aspect of quantitative analysis of isothiazolinones in environmental samples and human consumer products. The most common and widely applied sample preparation techniques include liquid–liquid extraction (LLE) and ultrasonication or solid-phase extraction (SPE). 

Liquid–liquid extraction remains one of the most powerful and versatile techniques in sample preparation for cleanup and enrichment, particularly for the analysis of organic compounds in aqueous solutions. LLE is also less expensive and flexible, as several samples may be prepared in parallel. Actually, many recent developments in LLE have been focused on new techniques, which greatly reduce the amount of solvent used (cost and environmental factors) and facilitate automation of the process in conjunction with chromatographic analysis [84]. In the literature, a number of reports describe the use of LLE during sample preparation of different samples prior to the evaluation of their isothiazolinone content [83,85,86]. 

Solid-phase extraction is one of the most common procedures for sample preparation in current use. SPE can be used for different purposes in sample preparation, such as cleanup, analyte concentration, and analyte derivatization. SPE offers a number of potential advantages over LLE, including higher analyte recoveries, no problems with emulsions, reduced organic solvent use, and requiring a smaller volume of sample. The wide variety of cartridge types and solvents makes the SPE procedure suitable for many polar or nonpolar analytes. For the analysis of isothiazolinones, different SPE stationary phases were used [83,86,87,88,89,90,91].

Nowadays, an increasing number of works report efficient sample preparation and extraction procedures based on SPE, pressurized liquid extraction (PLE), matrix solid-phase dispersion (MSPD), and advanced microextraction techniques such as solid-phase microextraction (SPME) and liquid–liquid microextraction (LLME), among others, to overcome the drawbacks arising from sample matrix interference (e.g., in cosmetics) [88,92,93]. Ultrasound-assisted extraction (UAE) can be used as an alternative to conventional sample preparation methods. Ultrasound is a key technology in achieving the objective of sustainable ‘‘green” chemistry and extraction. UAE has been employed for the extraction of isothiazolinones from solid and liquid samples with a high density and viscosity [87,89,90,94,95,96].

Combinations of these procedures with chromatographic techniques, coupled with different selective detectors, have been proposed as powerful tools to successfully analyze and determine the presence of isothiazolinones in different samples. In fact, most of the methodologies developed for the analytical determination involves the use of liquid chromatography (LC), high-performance liquid chromatography (HPLC), or, to a lesser extent, gas chromatography (GC). Table 5 summarizes the analytical methods reported in the literature for the determination of isothiazolinone biocides in different samples.

The use of GC does not show, in some cases, good performance for MI analysis, and in the case of BIT, it requires derivatization to improve its chromatographic performance. Rafoth et al. developed a GC-MS method for the determination of five isothiazolinones in environmental samples [83]. The results of the validation procedure showed that for CMI, BIT, OIT, and DCOI, the performance data were good and that the optimized method is well suited for their determination in environmental samples. Yet, for MI, the validation data was poorer and concluded that special care should be taken when using the developed method for the analysis of this compound, especially in wastewaters [83].

Methods based on LC or HPLC coupled to different detectors are the most used for the analysis of isothiazolinone biocides. The identification is carried out using several detectors, such as diode array (DAD) [89,99,102,108], simple quadrupole mass spectrometry (MS) [87,94], and triple quadrupole mass spectrometry (MS/MS) [85,88,90,91,93,95,101,104,106,107]. In general, MS/MS detector allows high sensitivity and selectivity, reducing the matrix interferences and increasing the signal-to-noise ratio. Ultra-performance liquid chromatography (UPLC) takes advantage of technological strides made in particle chemistry performance, system optimization, detector design, and data processing and control enabling dramatic increases in resolution, sensitivity, and speed of analysis, when compared with HPLC. Therefore, UPLC-MS/MS has been used as a powerful analytical technique for the determination of isothiazolinones in different matrices [85,88,90,95]. Wittenberg et al. developed and validated a UPLC–MS/MS method for the determination of MIT and MCI in cosmetic products. The proposed methodology was considered to be the fastest and most sensitive method for identifying and quantifying MI and MCI in cosmetic products [100]. Three of the seven products tested that contained both MI and MCI had MCI/MI ratios similar to that of Kathon CG (approximately 3:1), which seems to indicate that probably Kathon CG was the source of isothiazolinones for the preparation of the cosmetic products tested. The MCI/MI ratios for the other four products were calculated to be 2:1 or lower. The authors concluded that Kathon CG may not be the only source of these two preservatives for cosmetic formulations or that, alternatively, reactions of MCI and/or MI with other cosmetic ingredients within a given product may explain the unexpected ratios of MCI/MI observed [100].

## 6. Conclusions and Outlook

Isothiazolinone derivatives are synthetic preservatives that have been used for decades in a wide range of products, such as household detergents, water-based paints and glues, industrial biocides, and in cosmetics. Nowadays, isothiazolinones do represent, to a greater or lesser extent, potentially important health hazards, both for consumers and workers in numerous industries. In the EU, steps to restrict the use of some of these compounds in consumer products have been initiated, and stricter criteria for labeling and warning in chemical products have been approved.

Substantial efforts have been devoted to develop efficient methodologies to prepare isothiazolinones. However, most of the processes often still require the use of toxic and/or corrosive reagents. Therefore, the development of facile, efficient, and green synthetic methods to prepare isothiazolinones would be of prime value in order to maximize the desired products and minimize by-products that are environmentally and ecologically more benign.

One key parameter in the application of biocides is their stability. Chemical and photostability studies are an integral part of the product development process in order to ensure the quality, efficacy, and safety of the formulated products during manufacture, storage, and use and to understand the environmental fate of biocides. The literature data regarding isothiazolinone stability are still scarce; thus, more studies must be carried out to evaluate and understand their susceptibility to various chemical and biological transformation and degradation processes. The importance of a minimum amount of the right biocide to maintain long-term microbiological quality as well as the high cost of biocides makes accurate biocide quantification in the final product necessary. Furthermore, degradation products have been reported to be more toxic and/or persistent than the parent compounds, which highlights the need for the development of appropriate analytical detection methods for their determination and evaluation of their potential environmental impact.

Water-soluble biocides are prone to excessive leaching, and high concentrations are therefore required for the successful protection of products. Recent studies showed that nanotechnological encapsulation could be a solution for obtaining a longer-term protection from microbiological attack, prolonging the lifetime of biocides in different matrices by protecting them from leaching and by releasing them slowly. In this way, biocide encapsulation could be an environmentally friendly option, because the incorporation of high initial biocide concentrations to ensure long-term preservation would be avoided. Isothiazolinones encapsulation would allow maintaining a permanent release, preserving its biocidal activity even under conditions of temperature and pH at which their decomposition and hydrolysis occur. The controlled release of isothiazolinones could reduce environmental and human risks, improving the quality of the products. The encapsulation of biocides in suitable polymeric micro- and nanocarriers is a challenging and promising practice to restrict the leachability issue. The carrier can act as an intercepting barrier to biocide diffusion, enabling controlled release within the coating matrix and simultaneously the biocides that are unstable and prone to degradation can be protected from their environment via encapsulation. Currently, a rather limited number of studies regarding isothiazolinones encapsulation have been published, but the relatively recent launch on the market of antifouling products based on encapsulation technologies is an encouraging reason for further research on this field.

## Figures and Tables

**Table 1:** Minimum growth inhibitory concentration (MIC) values of MI, MCI, and BIT against *Schizosacchurornyces pombe* and *Escherichia coli*, as described by Collier et al. [30].

Biocides	MIC (µg/mL)	
	*S. pombe*	*E. coli*
MI	245	41
MCI	2.6	0.5
BIT	35.5	14.4

**Table 2:** MIC and minimum biocidal concentration (MBC) values for MI, MCI, MCI/MI, OIT, and DCOIT against *Aspergillus niger* and *Saccharomyces cerevisiae*. Values reported ± standard deviation of three to five replicates [33,34].

Biocides	*Aspergillus niger*		*Saccharomyces cerevisiae*	
	MIC (mg/L)	MBC (mg/L)	MIC (mg/L)	MBC (mg/L)
MI	166 ± 52	300 ± 0	60 ± 0	87.0 ± 10.3
MCI	0.35 ± 0.05	0.42 ± 0.04	0.58 ± 0.10	0.63 ± 0.10
MCI/MI	0.40 ± 0.07	0.47 ± 0.05	0.71 ± 0.10	0.76 ± 0.20
OIT	0.05 ± 0.01	0.05 ± 0.01	0.56 ± 0.10	0.65 ± 0.20
DOIT	0.12 ± 0.01	0.23 ± 0.14	0.55 ± 0.10	0.79 ± 0.20

**Table 3:** Calculated IC_50_ for MI, MCI, OIT, and DCOIT using glutathione reductase (GR) inhibition assay. Experimental LogP_ow_ and experimental EC_50_ observed in three cellular models (HepG2, *Vibrio fischeri*, and *Scenedesmus vacuolatus*) [57].

Biocides	GR Inhibition in HepG2 Cells IC_50_ (µM)	LogP_0W_	EC_50_ HepG2 (µM)	EC_50_ *V.fischeri* (µM)	EC_50_ *S.vacuolatus* (µM)
MI	>1000	−0.49	130	14	4.9
MCI	7.0	0.53	13	0.58	0.6
OIT	>1000	3.30	21	0.94	0.42
DCOIT	13.2	4.79	16	0.43	0.38

**Table 4:** Maximum concentration of isothiazolinones admitted in commercial products available in the EU [5,60].

Commercial Products	Maximum Admitted Concentration of Biocide
Cosmetics ^1^	MCI/MI < 15 ppm ^3^
	MI < 100 ppm ^4^
	BIT forbidden ^5^
	OIT forbidden ^6^
	DCOIT forbidden ^7^
Paints ^2^, glues ^2^, detergents ^1^	MCI/MI < 15 ppm
	MI < 300 ppm
	BIT < 360 ppm
	OIT insufficient data
	DCOIT insufficient data
Industrial biocides ^2^	MI, MCI, and BIT used at high concentrations (e.g., >5000 ppm)
	OIT insufficient data
	DCOIT insufficient data

^1^ Labeling is legally required; ^2^ No labeling required; ^3^ From April 2016 onwards: forbidden in leave-on cosmetics; ^4^ Banned from leave-on cosmetics since January 2017; ^5^ Exceptionally (illegally) presented in cosmetic in the EU market, allowed in cosmetic in the USS and Canada; ^6^ Exceptionally (illegally) presented in cosmetic in the EU market; ^7^ Exceptionally (illegally) presented in cosmetic in the EU market.

**Table 5:** Analytical methods for isothiazolinones determination in different products.

Analyte	Matrix	Sample Preparation	Analytical Technique	Reference
Sea-Nine 211 (DCOIT)	Marine sediments	FUSE/SPE clean-up; SBSE/TD pre-concentration step	GC-MS/MS	[97]
MCI, BIT, OIT, DCOIT	Water-based adhesive/Food contact material	Vortex extraction	HPLC-MS/MS	[98]
MI, MCI, BIT, OIT	Human hygiene consumer products	UAE	HPLC-MS	[87]
MI, MCI, BIT, OIT, DCOIT	Building and consumer products	SPE; ASE; UAE	GC-MS; UPLC-MS/MS	[88]
MI, MCI, BIT, OIT	Residential interior wall paints	LLE	UPLC-MS/MS	[85]
MI, MCI, BIT, OIT	Detergents and cosmetics	UAE	LC-MS	[94]
MI, MCI, BIT	Water-borne adhesives	SLE	HPLC-DAD	[99]
MI, MCI	Cosmetic products	Mechanical shaking	UPLC-MS/MS	[100]
MI, MCI, BIT, OIT, DCOIT	Polyvinyl alcohol cooling towels	Mechanical shaking	LC-MS/MS	[101]
MI, MCI	Adhesives for food packaging	HF-LPME	HPLC-DAD	[102]
MI, MCI	Cosmetics, cleaning agents, pharmaceuticals	UAE	UPLC-DAD	[103]
MI, MCI	Cosmetics, pharmaceuticals	UAE	HPLC-DAD	[89]
MI, MCI, BIT, OIT	Cosmetics and household products	MSPD	HPLC-MS/MS	[93]
MI, CMI, OIT	Gel-products used for cooling	Mechanical shaking	LC-MS/MS	[104]
BIT, OIT, DCOIT	Surface water, wastewater, sediment, sludge, soil	SPE, UAE	UPLC-MS/MS	[90]
MI, MCI	Cosmetic products	UAE	HPLC-DAD	[105]
MI MCI, DCOIT	Drinking waters, surface waters, wastewater	Filtration	HPLC-MS/MS	[106]
MI, MCI, BIT, OIT	Paper used for food packaging	UAE	UPLC-MS/MS	[95]
MI, MCI, BIT, OIT, DCOIT	Facade run-off waters	SPE	HPLC-MS/MS	[91]
BIT, OIT	Wastewater, river water	SPE	LC-MS/MS	[107]
MI, MCI	Cosmetics	UAE	UPLC-DAD	[96]
MI, MCI, BIT, OIT, DCOI	Surface waters, groundwater, drinking water	LLE, SPE	GC-MS	[83]
MI, MCI, BIT, OIT	Non-formalin adhesives	LLE, SPE	GC-MS	[86]
MI, MCI	Cosmetics	Separation and purification by column chromatography	HPLC-DAD	[108]

FUSE, focused ultrasound-assisted extraction; SLE, solid-liquid extraction; SPE, solid-phase extraction; SBSE, stir bar sorptive extraction; TD, thermal desorption system; UAE, ultrasonic-assisted extraction; ASE, accelerated solvent extraction; LLE, liquid–liquid extraction; HF-LPME, three-phase hollow-fiber liquid-phase microextraction; MSPD, matrix solid-phase dispersion.
